# Genetic Association Between Androgen Receptor Gene CAG Repeat Length Polymorphism and Male Infertility: A Meta-Analysis

**DOI:** 10.1097/MD.0000000000002878

**Published:** 2016-03-11

**Authors:** Bihui Pan, Rui Li, Yao Chen, Qiuqin Tang, Wei Wu, Liping Chen, Chuncheng Lu, Feng Pan, Hongjuan Ding, Yankai Xia, Lingqing Hu, Daozhen Chen, Jiahao Sha, Xinru Wang

**Affiliations:** From the State Key Laboratory of Reproductive Medicine (BP, RL, YC, WW, CL, YX, XW), Institute of Toxicology, School of Public Health; Key Laboratory of Modern Toxicology of Ministry of Education (BP, RL, YC, WW, CL, YX, XW), Nanjing Medical University; State Key Laboratory of Reproductive Medicine (QT, HD), Department of Obstetrics, Nanjing Maternity and Child Health Care Hospital Affiliated to Nanjing Medical University, Nanjing; State Key Laboratory of Reproductive Medicine (WW, LH, DC), Wuxi Maternal and Child Health Care Hospital Affiliated to Nanjing Medical University, Wuxi; Department of Gynecology and Obstetrics (LC), The Second Affiliated Hospital of Nantong University, Nantong; State Key Laboratory of Reproductive Medicine (FP), Department of Andrology, Nanjing Maternity and Child Health Care Hospital Affiliated to Nanjing Medical University; and State Key Laboratory of Reproductive Medicine (JS), Nanjing Medical University, Nanjing, China.

## Abstract

The association between polymorphism of androgen receptor gene CAG (AR-CAG) and male infertility in several studies was controversial. Based on studies on association between AR-CAG repeat length and male infertility in recent years, an updated meta-analysis is needed. We aimed to evaluate the association between AR-CAG repeat length and male infertility in advantage of the data in all published reports.

We searched for reports published before August 2015 using PubMed, CNKI, VIP, and WanFang. Data on sample size, mean, and standard deviation (SD) of AR-CAG repeat length were extracted independently by 3 investigators.

Forty-four reports were selected based on criteria. The overall infertile patients and azoospermic patients were found to have longer AR-CAG repeat length (standard mean difference (SMD) = 0.19, 95% confidence interval (CI): 0.10–0.28, *P* *<* 0.01; SMD = 0.36, 95% CI: 0.10–0.61, *P* *<* 0.01). AR-CAG repeat length was longer in infertile men in Asian, Caucasian, and mixed races (SMD = 0.25, 95% CI: 0.08–0.43, *P* *<*0.01; SMD = 0.13, 95% CI: 0.02–0.25, *P* *<*0.05; SMD = 0.39, 95% CI: 0.15–0.63, *P* *<*0.01). The overall study shows that increased AR-CAG repeat length was associated with male infertility. The subgroup study on races shows that increased AR-CAG repeat length was associated with male infertility in Asian, Caucasian, and mixed races. Increased AR-CAG repeat length was also associated with azoospermia.

This meta-analysis supports that increased androgen receptor CAG length is capable of causing male infertility susceptibility.

## Abstract 2

Supplemental Digital Content is available in the text

## INTRODUCTION

Infertility has become a threat to more and more couples and aroused lots of attention during recent years. About 50% infertile cases are attributed to male factor. Nevertheless, the etiology of half of male infertility cases remains obscure.^1^ Many factors potentially compromising male reproductive ability have been searched, postulated, and studied.

Androgen, mainly secreted by Leydig cells in male testis, is essential for male sex differentiation, development, spermatogenesis, and sexual behavior. It is mediated by the androgen receptor (AR), a member of the steroid hormone receptor superfamily. The gene of AR is located at chromosome Xq11-12, which has 8 exons and 7 introns.^2^ The protein encoded by exon 1 of the AR gene is linked to AR transcriptional activity.^3^ The exon 1 of AR gene contains CAG and GGC trinucleotide polymorphic repeats which can respectively encode for polyglutamine and polyglycine. Previous studies have reported that the lengths of the 2 polymorphisms vary in different people.^4–6^ The usual variation of the AR-CAG trinucleotide repeat length is 11 to 36,^7^ and the median number is 22 in Caucasians.^8^ AR-CAG trinucleotide repeats were found possibly in association with male fertility in 1991 for the first time,^9^ and a great many studies followed to further that investigation. But the results are divergent.

Some studies demonstrated that longer length of AR-CAG trinucleotide repeats was associated with increased risk of male infertility, which is typical of impaired spermatogenesis with different severity. Also, it was suggested that AR-CAG tracts longer than 40 repeats give rise to Kennedy disease, a fatal neuromuscular disease accompanied by reduced sperm quality.^10^ Other reports, however, did not provide corresponding results. Whether AR-CAG trinucleotide repeats is linked to male infertility has not been fully revealed. We hope that an updated meta-analysis based on the data obtained from recent studies and all published literature could be helpful to the deeper exploration into this field.

## METHODS

### Study Design

Our study was composed of searching eligible reports, extracting data, analyzing data, and conducting sensitivity analysis and testing publication bias. We classified infertile men into different groups according their ethnic races and sperm concentration in hopes of exploring more potential factors which lead to male infertility. Ethical approval was waived because this study is a meta-analysis.

### Searching Strategy

To link AR-CAG trinucleotide repeat length and risk of male infertility, we searched the PubMed, China National Knowledge Infrastructure (CNKI), VIP Database for Chinese Technical Periodicals (VIP), WanFang Med Database (WanFang) to retrieve all articles available before August 2015 without language restrictions. The keywords used were a combination of “AR, CAG, male infertility.’. All reports were independently identified by 2 authors, and references cited in all original reports and review articles were examined through manual retrieval to identify other potentially eligible publications.

### Inclusion Criteria and Exclusion Criteria

**FIGURE 1:**
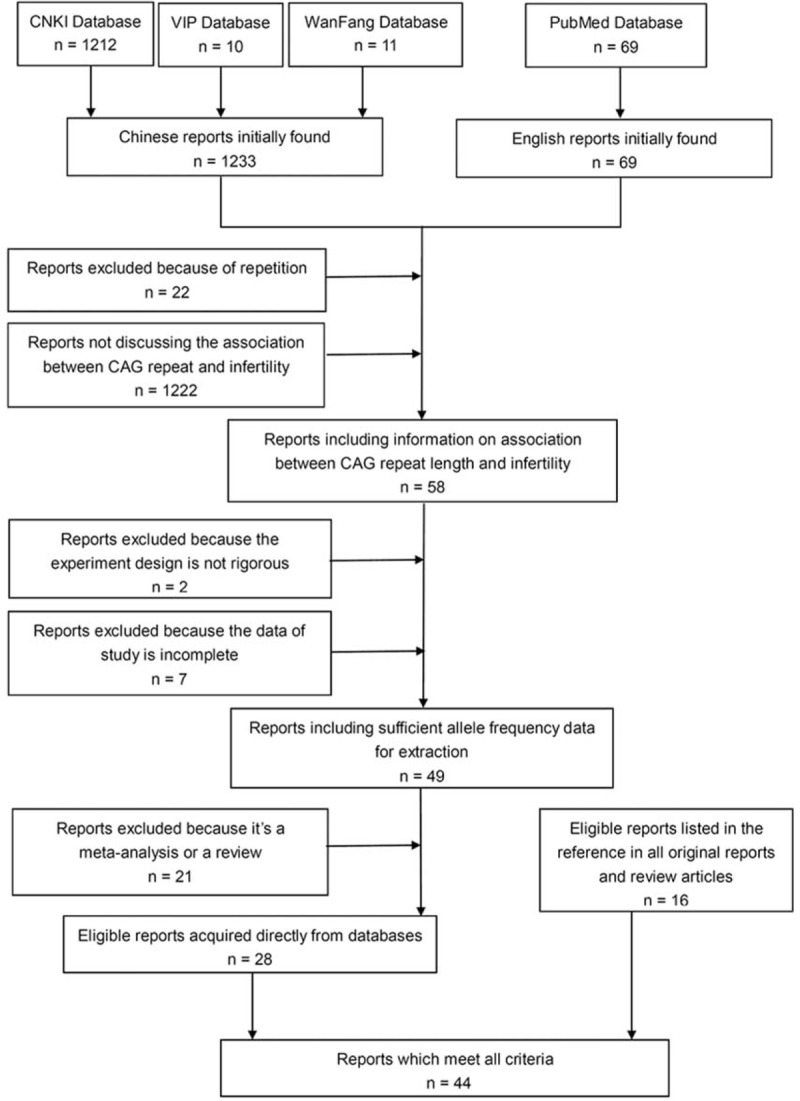
Flow diagram of the study selection process.

The included studies should be the ones that evaluates the association between AR-CAG trinucleotide repeat length and risk of male infertility, includes a case group of male patients with male infertility, which some of them were divided into azoospermia group and oligospermia group by semen analysis according to World Health Organization guidelines^11^, includes a control group of proven or presumed fertile men, and includes sufficient allele frequency data for extraction. Studies were excluded because the design of the study is not rigorous, data of study is incomplete, repetition of the published literature, and a meta-analysis or a review. The procedure of articles screening is illustrated in a flow chart (Figure 1). Data from those reports were extracted and summarized in Table 1. Some reports not included in this meta-analysis but of reference value are also shown in Table 1 (marked by superscript *a*). All reports collected in Table 1 were listed according to its date of publication.^1,12–65^

**TABLE 1:**
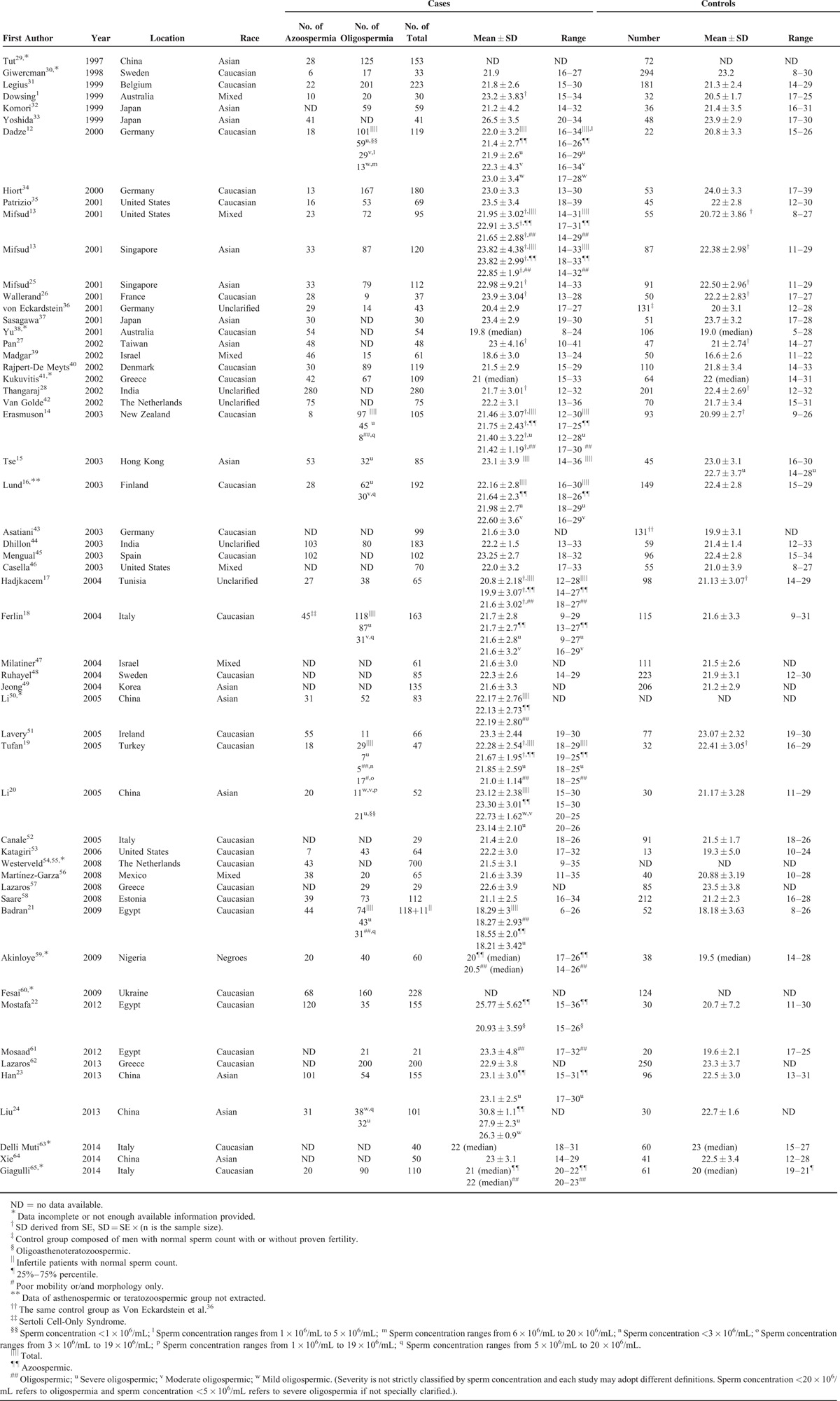
A Summary of Studies on Association Between Male Infertility and CAG Repeat Length in Androgen Receptor Gene

### Data Extraction

All eligible reports were screened independently by 3 investigators (BP, RL, and YC) to extract available data according to the prespecified selection criteria. Disagreement was resolved by discussion with coauthors. The sample size, mean, and standard deviation (SD) of case and control group were extracted. In some studies,^12–24^ azoospermic and oligospermic groups were specially classified and the data of each group were respectively extracted. Information of the range of AR-CAG repeat length, geographic location of the study, year of publication, ethnicity of participants was also noted. In several articles,^1,13,14,17,19,25–28^ we derived *SD* from standard error (*SE*): *SD* = *SE* × *n*, where n represents the size of case or control groups.

### Data Analysis

All statistical analyses were carried out using Stata (Version 9.2, StataCorp LP, College Station, TX), and *P* <0.05 were considered to be significant. Adding to the study on the total infertile case group, we conducted studies on the group of azoospermia and oligospermia. When it comes to oligospermic group, we classified it into the mild oligospermic group (sperm concentration >5 × 10^6^ and <20 × 10^6^/mL) and severe oligospermic group (sperm concentration <5 × 10^6^/mL). In some studies, the definition of mild oligospermia and severe oligospermia was different from other reports.^12,19,20^ Since the difference was not too wide to influence the study, so the data of those reports were also included in the study of mild or severe oligospermia. To explore the effect of race of study participants on results, we also conducted studies based on the race (Caucasian, Asian, Mixed races, and Unclarified race). The standardized mean difference (SMD) of the AR-CAG repeats length and its 95% confidence interval (95% CI) were used as statistic. Homogeneity of the included studies was tested. When *P* <0.1 or *I*^2^ >50%, there is a high extent of heterogeneity between studies and random-effect model was used; when *P* *>*0.1 and *I*^2^ <50%, no heterogeneity is found between studies, so fixed-effect model was used. The impact of quality of the included studies on the results was estimated by sensitivity analysis. Funnel plot studies were used to evaluate the publication bias.

## RESULTS

### Searching Results

We selected articles in accordance with the process demonstrated in Figure 1. Eventually, 41 English and 3 Chinese reports meet all criteria of inclusion.^1,12–28,31–37,39,40,42–49,51–53,56–58,61,62,64^ The characteristics of the selected studies are shown in Table 1. One report^25^ included studies on 2 different ethnic groups, so we consider it as 2 separate studies. Three studies^22–24^ did not provide data for the overall infertile patients. They only provided the data for subgroups such as azoospermic group or oligospermic group. So we did not include these 3 studies in our analysis for the overall study, but they were included in studies of subgroups. Data of azoospermic group were provided by 19 reports.^12–24,27,28,33,37,42,45^ Six reports^13,17,25,32,61,62^ provided data of oligospermic group without further stratification. Some reports^12,14–16,18-21,23,24^ classify oligospermic group according to sperm concentration. We got 3950 cases and 3684 controls for the overall study. At the same time, we got 1145 cases and 1447 controls for the study of azoospermic group, 442 cases and 664 controls for the study of severe oligospermic group, as well as 210 cases and 523 controls for the study of mild oligospermic group.

### Association Between AR-CAG Repeat Length and Male Infertility

**FIGURE 2:**
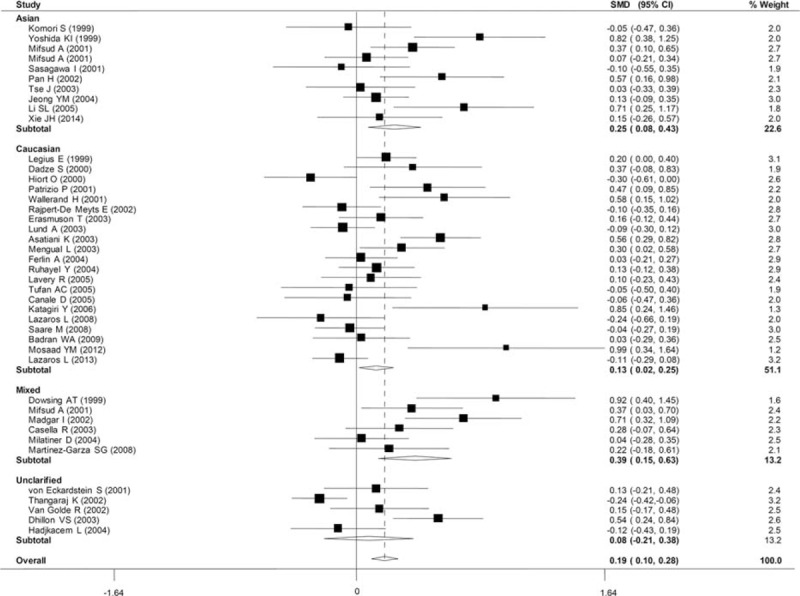
Forest plot of *AR* CAG repeat length polymorphism and male infertility risk. The horizontal line represents the 95% confidential interval. The length shows the range of the confidential interval and the size of the square in the middle shows the weight of the study. The diamond (and broken line) represents the overall summary estimate, with confidence interval given by its width. The unbroken vertical line is at the null value (OR = 1.0). CI = confidence interval; SMD = standardized mean difference.

**FIGURE 3:**
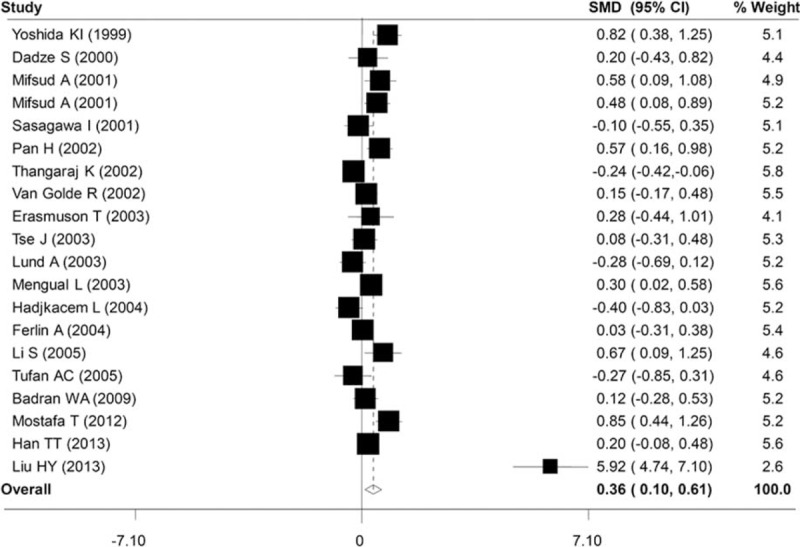
Forest plot of *AR* CAG repeat length polymorphism and azoospermia risk. The diamond (and broken line) represents the overall summary estimate, with confidence interval given by its width. The unbroken vertical line is at the null value (OR = 1.0). CI = confidence interval; SMD = standardized mean difference.

The results of this meta-analysis on the association between AR-CAG repeat length polymorphism and male infertility are illustrated in Table 2. The overall meta-analysis between case and control group revealed significant difference in the length of AR-CAG repeat length polymorphism (SMD = 0.19, 95% CI: 0.10–0.28, *P* *<*0.001) (Figure 2). The results of subgroup study on the patients’ races showed that longer AR-CAG repeat length was associated with male infertility in Asian, Caucasian, and population of mixed races (SMD = 0.25, 95% CI: 0.08–0.43, *P* *<*0.01; SMD = 0.13, 95% CI: 0.02–0.25, *P* *<*0.05; SMD = 0.39, 95% CI: 0.15–0.63, *P* *<*0.01) (Figure 2). However, in some studies that did not specify the races of study population, no notably difference was observed in the AR-CAG repeat length between case and control groups (SMD = 0.08, 95% CI: −0.21–0.38, *P* *>*0.05) (Figure 2). Combined data of azoospermic group revealed that AR-CAG repeat length of case group was positively longer than that of control group (SMD = 0.36, 95% CI: 0.10–0.61, *P* *<*0.01) (Figure 3). However, AR-CAG repeat length was not significantly longer than that of control group in both severe oligozoospermic groups and mild oligospermic group (SMD = 0.32, 95% CI: −0.33–0.66, *P* *>*0.05; SMD = 0.47, 95% CI: −0.11–1.06, *P* *>*0.05) (Figures S1 and S2).

**TABLE 2:**
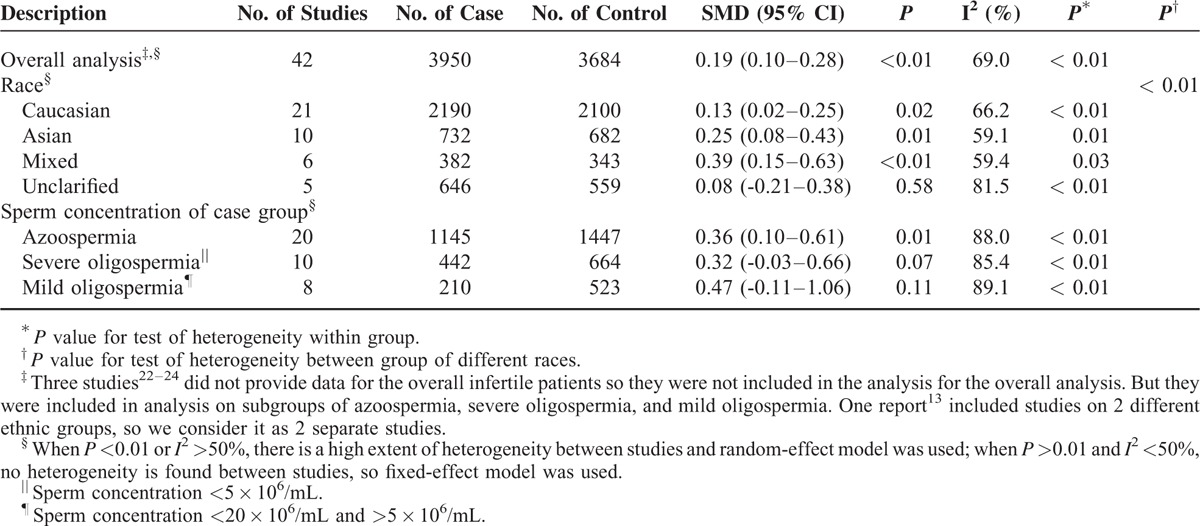
Main Results of *AR* Repeat Length Polymorphism in the Meta-Analysis

### Sensitivity Analysis and Evaluation of Publication Bias

Sensitivity analysis was conducted by wiping out 1 report each time to find whether the result of the study would be changed. Results remained the same in analysis on overall infertile men, Asian race, mixed race, unclarified race, azoospermic group, and mild oligospermic group. In the analysis on Caucasian race, the result was changed by wiping out 1 of the reports.^43^ The study showed no association between AR-CAG repeat length and male infertility. In severe oligospermic group, the result showed that increased AR-CAG repeat length could be a risk for male infertility after wiping out 2 of the reports.^16,19^ These results may be caused by different inclusion standard and number of cases. No obvious publication bias was found based on the shape of funnel plot studies (Figure S3).

## DISCUSSION

Spermatogenesis disorder has a complex pathogenesis. Androgen functions through combining with AR to stimulate or inhibit the expression of relevant gene. The number of glutamine encoded by (CAG)n is essential to the structure and function of AR molecule and its cofactors. The normal range of CAG repeat length is considered between 16 and 29.^66^

Reports on association between male infertility and gene polymorphism of androgen receptor mostly focused on Caucasian race and Asian race. Our study suggests that Asian men are more easily affected by the abnormality of AR-CAG polymorphism. Our study shows that the overall infertile population is typical of an increased AR-CAG repeat length. When it comes to racial factors, Asian, Caucasian, and mixed races all show the same result. In recent years, many studies classified infertile patients according to sperm concentration, such as azoospermia and oligospermia. We conducted analysis on those groups to explore the possible relationship between the severity of defect spermatogenesis and AR-CAG repeat length. Azoospermia was found to be associated with increased AR-CAG repeat length, but this result was not found in either severe oligospermia or mild oligospermia. That indicates oligospermia could be a result of many more complex factors and increased AR-CAG repeat length could result in severe spermatogenesis disorder. The study of Liu et al^24^ included in the meta-analysis is different from others. Liu et al^24^ using small sample size found that the AR-CAG repeat length was 30.8 ± 1.1 in group of azoospermia, which was significantly higher than that of fertile control. The study with a relatively small sample size might lack of adequate power to draw a fair conclusion. Additionally, the gene polymorphism might differ among different geography areas and race. Therefore, future studies with larger sample size in this area are needed to verify the association between AR-CAG repeat length and male infertility. Though the result of the study was different from others, it also met the strict criteria of study and thus were included in our meta-analysis.

(GGN)n polymorphism has also been analyzed but its function is still unknown. Nevertheless, many more studies revealed that there was no association between AR-GGN repeat length and male infertility.^18,59,67^ When the joint of CAG and GGC was taken into consideration, 2 haplotypes (CAG = 21/ GGC = 18, CAG ≥21/GGC ≥18) could make the risk of male infertility increase.^18^ Whether (GGN)n polymorphism could influence the function of androgen receptor remains to be fully studied.

Meta-analysis is a quantitative systematic review and its result could be influenced by publication bias, database bias, inclusion criteria bias, and language bias. We followed the strict criteria to eliminate the ineligible reports to ensure the quality of included studies. Funnel plot studies were conducted. We searched both PubMed database and Chinese database such as CHKI, VIP, WanFang to get a more comprehensive set of data.

AR-CAG repeat polymorphism has been studied for its influence on decreased sexual function, which could lead to infertility. Increased CAG repeat length was found to compromise sexual function.^68^ Nevertheless, no significant association was found between erectile dysfunction and CAG repeat length.^69^ Increased CAG repeat was also found to be associated with depression and men with CAG repeat length ≥23 more frequently encountered decreased potency.^70^ Recent studies show that hypogonadal patients with shorter CAG repeat length had a more significant improvement after testosterone replacement therapy.^71,72^ Those reports indicated that failed sexual intercourse resulting from impaired sexual function could be a reason why increased CAG repeat length could lead to male infertility. Additionally, our meta-analysis suggests that AR-CAG repeat polymorphism has a relationship with male infertility, but the exact molecular mechanisms of how the CAG repeat polymorphism affects male infertility are unknown. We suppose that the secondary structure of AR mRNA sequence or the transcription factors binding site may change by the longer CAG repeat length. Our analysis suggests that further exploration of the molecular mechanism of AR-CAG repeat polymorphism and risk male infertility is demanded.

The present study had some limitations that require consideration. First, some studies with small sample size may not have enough statistical power to explore the real association. Second, our results were based on unadjusted estimates and a more precise analysis should be conducted if individual data were available, which would allow for adjustment by other covariants such as age, body mass index, smoking status, drinking status, environment factors, and lifestyle. Third, in the subgroup analyses, the number of mild oligospermia was relatively small, not having enough statistical power to investigate the association of the polymorphism with male infertility susceptibility.

In conclusion, we again confirmed the association between increased AR-CAG repeat length and defected spermatogenesis. However, its association with the severity of the disease is to be fully studied. The studies on other possible factors such as race and lifestyle are also needed. AR-CAG polymorphism could be an effective way of evaluating the risk of infertility. The studies also paved the way for the gene therapy for the male infertility. Further studies are needed to clarify the association between length of CAG repeats and male infertility as well as its mechanism.

## Supplementary Material

Supplemental Digital Content
