# Supplementary material for: Genetic Association Between Androgen Receptor Gene CAG Repeat Length Polymorphism and Male Infertility: A Meta-Analysis
Source: Medicine (Baltimore). 2016 Mar 11;95(10):e2878. doi: 10.1097/MD.0000000000002878 (PMC4998865; doi:10.1097/MD.0000000000002878)
Supplement: Supplemental Digital Content [file medi-95-e02878-s001.doc]

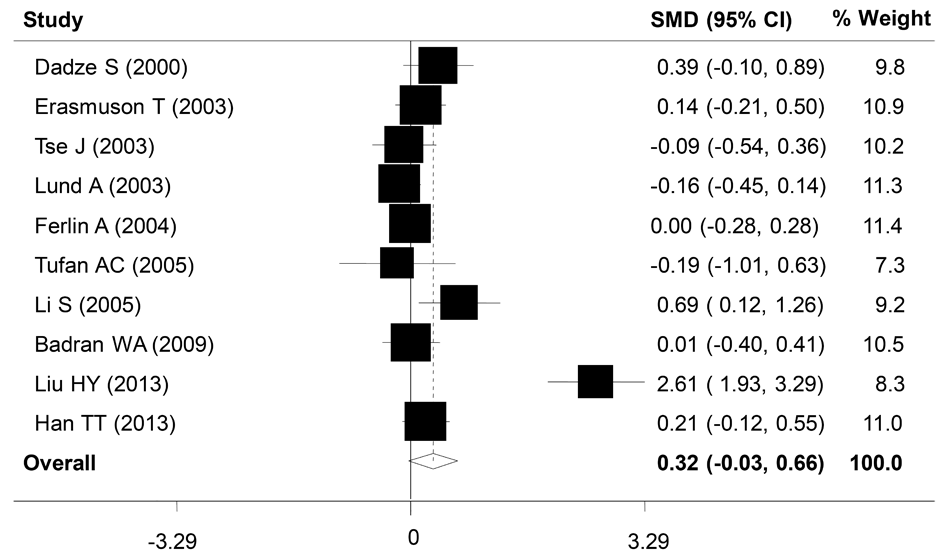


Figure S1 Forest plot of *AR* CAG repeat length polymorphism and severe oligospermia risk. The diamond (and broken line) represents the overall summary estimate, with confidence interval given by its width. The unbroken vertical line is at the null value (OR░=░1.0). SMD, standardized mean difference; CI, confidence interval.


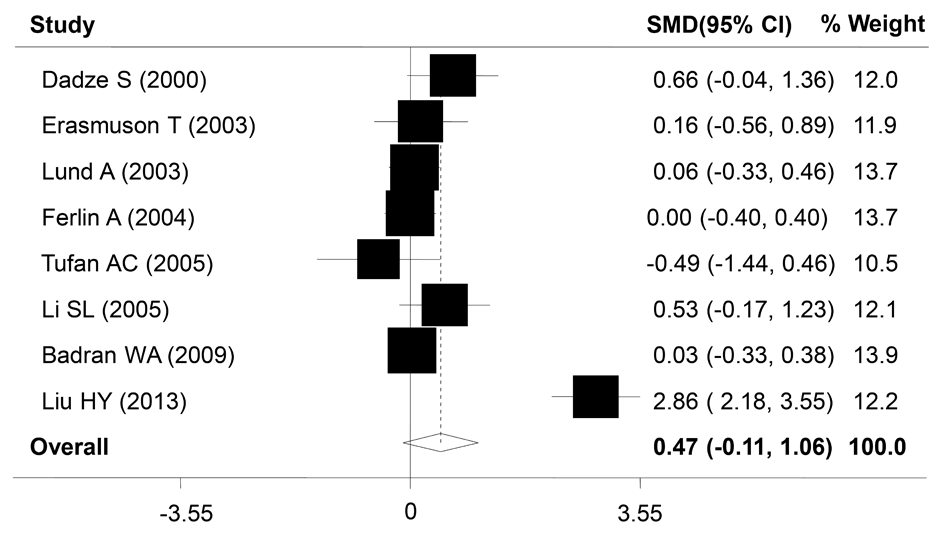


Figure S2 Forest plot of *AR* CAG repeat length polymorphism and mild oligospermia risk. The diamond (and broken line) represents the overall summary estimate, with confidence interval given by its width. The unbroken vertical line is at the null value (OR░=░1.0). SMD, standardized mean difference; CI, confidence interval.


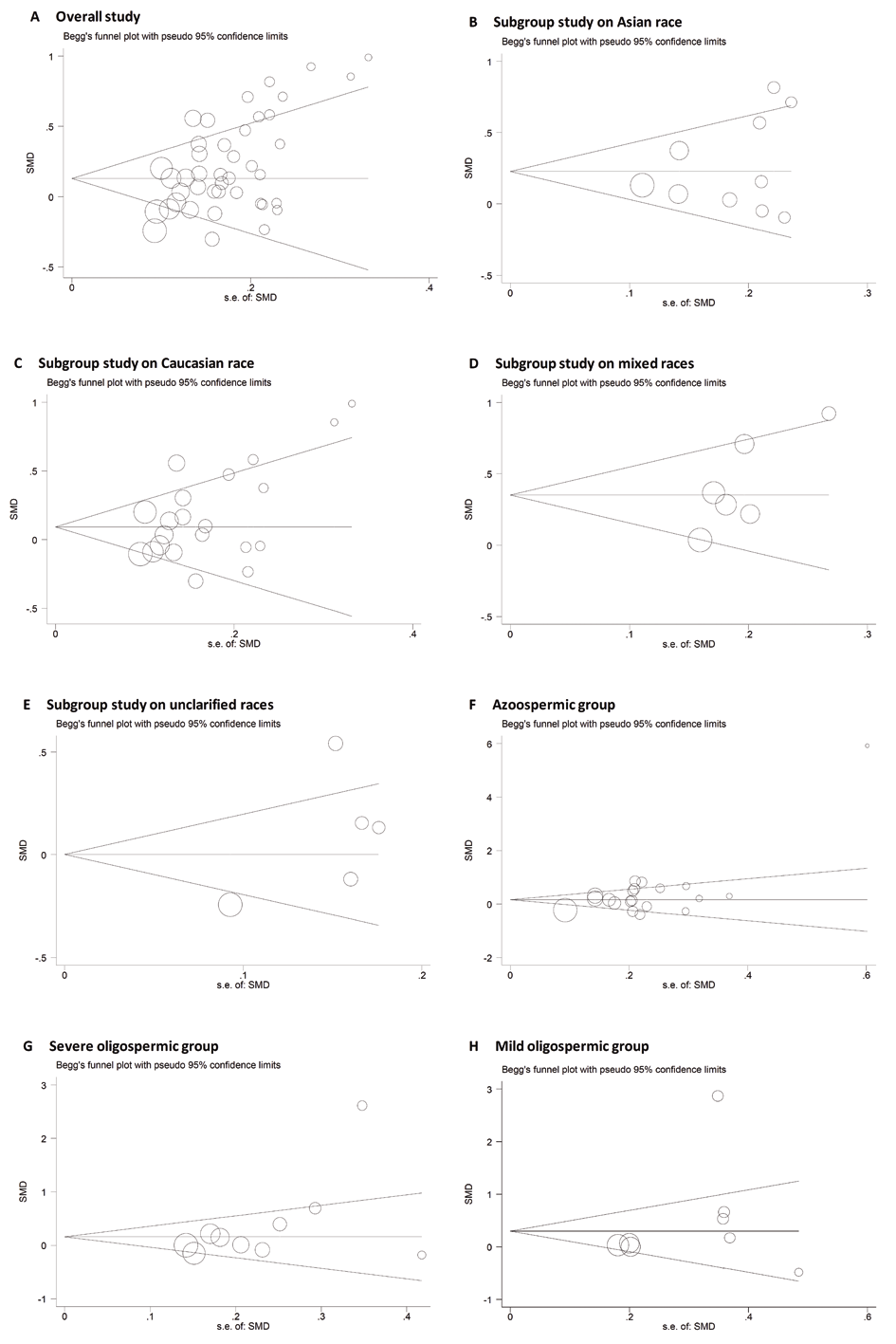


Figure S3 Funnel plot analysis to detect publication bias. Each point represents a separate study for the indicated association. (A) Funnel plot for *AR* CAG repeat length polymorphism in overall analysis. (B) Funnel plot for *AR* CAG repeat length polymorphism in subgroup of Asian race analysis. (C) Funnel plot for *AR* CAG repeat length polymorphism in subgroup of Caucasian race analysis. (D) Funnel plot for *AR* CAG repeat length polymorphism in subgroup of mixed race analysis. (E) Funnel plot for *AR* CAG repeat length polymorphism in subgroup of unclarified race analysis. (F) Funnel plot for *AR* CAG repeat length polymorphism in overall analysis. (G) Funnel plot for *AR* CAG repeat length polymorphism in azoospermia analysis. (H) Funnel plot for *AR* CAG repeat length polymorphism in severe oligospermia analysis. (I) Funnel plot for *AR* CAG repeat length polymorphism in mild oligospermia analysis.
